# Caregivers’ Burden in Patients With Bipolar Disorder and Schizophrenia and Its Relationship With Anxiety and Depression in Caregivers: A Narrative Review

**DOI:** 10.7759/cureus.47497

**Published:** 2023-10-22

**Authors:** Anshita Girdhar, Ragini Patil

**Affiliations:** 1 Psychiatry, Jawaharlal Nehru Medical College, Datta Meghe Institute of Higher Education and Research, Wardha, IND

**Keywords:** mental illness, depression, schizophrenia, qol, bpd

## Abstract

Mental disorders affect a person's thinking, mood, and/or behaviour and can range in severity from minor to severe. Nearly one in five persons have a mental disease as stated by the National Institute of Mental Health. A serious mental illness called bipolar disorder causes extreme mood swings that can range from manic to depressive states. Schizophrenia is a brain condition that leads individuals to perceive reality differently. They cannot distinguish between what they are actually experiencing and what they are just imagining. Both illnesses have a variety of negative effects on the patient as well as the primary caregiver, who may be the patient's family or other relatives. In the case of a patient with mental illness, the family's role is crucial. Family members' long-term caregiving obligations may result in a caregiving burden that negatively impacts the caregivers' quality of life, career and personal relationships. Depression generally undermines carers' ability to fulfil their crucial supportive role towards their relative with a mental illness while contributing to their distress and handicap. Given the high prevalence of caregiver depression, it is critical to address this issue not just by creating therapies to treat caregiver depression once it has started, but also by preventing caregiver depression.

## Introduction and background

Schizophrenia (SCZ) is a severe and chronic mental illness that affects over 20 million individuals worldwide. It is a serious condition that impairs the patient's intellect, perceptions, and behaviours to varying degrees [1]. One of the most prevalent mental conditions, bipolar affective disorder (BPD), has a lifetime prevalence of roughly 3% in the general population and is the sixth greatest cause of disability globally [2]. This illness is characterised by recurrent episodes of the patient's mood and activity levels being drastically out of balance [3]. Ogilvie et al. discovered that BPD worsens the quality of life (QOL), reduces productivity at work, and leads to chronic impairment. BPD has a notable ruinous effect on members of the family and caregivers' QOL, besides putting people/patients through pain. The prevalence of BPD, a recurring, severe mental illness, ranges from 1.3% to 3.8% [4]. The functioning of the entire family can be negatively impacted by a family member's chronic illness, especially if it is a serious mental illness, particularly in terms of work achievement and social or recreational activities. Due to the statistically substantial impairments brought on by their illness, patients with SCZ and BPD are frequently dependent on their caretakers. Families are anticipated to provide the majority of the assistance and care for the mentally ill family member for a duration of time that is statistically significant while being constantly concerned about relapse and how the sickness may affect other family members [5]. It is acknowledged that having a family member with a mental illness and caring for them puts a strain on the family. There are two distinct sorts of load: subjective burden, which refers to an individual's opinion of the situation as oppressive; and objective burden, which refers to the obvious costs to the family that come from the condition [6,7]. The rising frequency of psychiatric issues and the gradual deinstitutionalisation of their care may put a lot of stress on the caregivers. Additionally, in order to prevent burnout and maintain their own mental health, caregivers need social support [8,9]. Numerous research has been carried out worldwide, including in India, to determine how the burden of care varies depending on the demographic type. Studies have shown that those who care for people with psychiatric illnesses experience more burdens than those who care for people with other chronic medical conditions [10-13]. We conducted this review to assess the burden and its connection to depression and QOL in those who provide care for patients with SCZ and BPD.

## Review

Burden among caregivers

Caregivers face difficulties when caring for patients with serious psychiatric disorders like SCZ and BPD. Concern over their capacity to manage or cope with the growing expectations and obligations has increased [14]. According to a cross-sectional study conducted by Walke et al., which included 320 caretakers of mentally unstable individuals and assessed the caregivers' burden and coping mechanisms, severe caregiver burden accounted for 40.9% of all caregivers' burdens, while 59.1% of caregivers experienced a moderate level of burden. This is equivalent to the research done by Mandal et al. among 30 carers of patients with SCZ. In another study including 100 caretakers of SCZ patients, Kaur et al. in Punjab also found comparable results, with 50% experiencing a moderate load and 49% experiencing a severe one [15-17]. The burden assessment scale used in the Walke et al. study also revealed that the highest levels of burden were observed in the domains of mental and physical health, which evaluated the stress of caregiving brought on by feelings of fatigue, frustration, and depression, as well as the burden in areas requiring outside assistance, such as encouragement and gratitude from loved ones, friends, and family. The patient's support, including the need for help, and other relationships, including the interruption of family activities and effects on other relationships, carried the least hardship [18].

A multicentre cross-sectional study that evaluated the anxiety, QOL, depression, and self-esteem of caregivers of individuals with SCZ, major depressive disorder, and BPD found that they had the lowest QOL in comparison to those who cared for people with BPD [19]. Due to various circumstances, including social isolation and stigma, caregivers are likely to encounter sadness and anxiety owing to their caregiving responsibilities. On the other hand, worrying and pessimistic thought patterns may cause caretakers to concentrate more on the unpleasant aspects of life. Inevitably, the burden of care may lower QOL by causing despair and worry [20].

In a cross-sectional study with 97 members of the family of mentally ill patients, it was determined that 40.2% had a mild burden, 27.8% had a moderate burden, 25.8% had less or no burden, and 6.2% had a severe burden [21]. This result is in accordance with the findings of Bhandari et al., who found that 48.2% of caregivers experienced a mild level of burden [22]. In another study conducted by Shamsaei et al., 225 family caregivers were chosen, and the burden of the caregiver was evaluated. The results showed that a mild to moderate burden was seen in 41.8% of caregivers, 27.1% had a severe burden, and 59.2% had a moderate burden [23]. Pun et al.'s research in Nepal revealed that 46.9% of relatives giving care felt a moderate amount of burden, and a substantial load was experienced by family caregivers in 52% of cases. The study's environment and different sample sizes could be to blame for the inconsistent results [24].

A study in the United States found that individuals with higher caregiver burdens had shorter education experiences. This may be because caregivers with greater levels of education have a better understanding of the complexities of caregiving, and they may also have defined roles and responsibilities. In contrast, caregivers who were caring for patients with only a rudimentary education faced more difficulties. A prior study that found a higher caregiver burden was linked to a lower education level validated this. High levels of burden can result from patients' lower productivity and earning capacity during their remission period. Additionally, informed individuals may be more aware of their condition and seek assistance and treatment earlier, reducing the strain on relatives giving care to sufferers [25-28].

SCZ patient caregivers bear a heavier burden

In order to compare the type and level of load felt by relatives of individuals with SCZ and BPD, Vasudeva et al. studied 52 patients with SCZ and 51 patients with BPD. Those with BPD had a substantially longer median disease duration than those with SCZ (P < 0.05), but patients with SCZ had a significantly longer median untreated illness duration. The average age of the caregivers was 48.32 years for the bipolar group and 47.39 years for the SCZ group. The average age of the caretakers was 48.32 years for those with SCZ and 47.39 years for those with BPD, respectively. In the domains of external assistance (P < 0.05, Cohen's d = 0.479), caregiver routine (P < 0.01), and other relations (P < 0.01), caregivers of SCZ patients reported considerably higher load [29].

The culpability to look after mentally unhealthy individuals can be challenging, particularly when the patient has a chronic illness like SCZ or BPD when they become detached from reality. In a cross-sectional study, it was determined how much work it was for caregivers to take care of patients with SCZ and BPD and how much work was different for each condition. The study involved 210 caregivers and was interview-based. The average burden score in the SCZ and BPD groups, respectively, was 64.89 ± 15.7 and 59.11 ± 17.8, and the dissimilarity among the groups was statistically remarkable, according to the authors. It was discovered that the caregivers' load greatly increased in both groups as patients' ages grew [30].

In a cross-sectional study by Khatoon et al. on 30 patients with SCZ and BPD to compare the pattern of burden among families, they discovered that the family of SCZ patients have a remarkably greater load than the relatives of BPD sufferers with respect to financial load, interruption of family activities at home, total burden [31].

In a similar study with 200 caregivers of sufferers with SCZ and BPD, it was discovered that the level of burden was less in the BPD group in contrast to the caregivers of individuals with SCZ. The total score of burden for the caregivers of BPD was considerably lower compared to caregivers with SCZ (t = 5.2). In comparison to the BPD group, QOL in caregivers was considerably lower in the SCZ. The highly notable negative association amidst caregiver QOL, load in both groups of caregivers demonstrate the difficult effects of this activity on the family members of psychologically ill person [32].

This result is consistent with a study that looked at 125 caregivers in south Brazil and found that those with SCZ had poorer QOL scores than those caring for BPD patients. Significantly poorer QOL scores were correlated with the presence of clinical disease, female gender, schizophrenia diagnosis in the patient, and the frequency of depressive symptoms in the caregiver. Their research also showed that caregivers' QOL levels were lower than those of the general Brazilian population [33].

No significant difference

A cross-sectional investigation was undertaken by Abbaslou et al. with 100 caregivers of individuals with SCZ or bipolar I disorder to assess caregiver burdens and coping mechanisms. They discovered an inverse relationship between burden and coping mechanisms for caregivers in the SCZ and BPD groups (P < 0.01). Two separate t-tests on samples revealed that the load among caregivers of patients with SCZ and BPD was not different (P < 0.24), despite a direct association between burden and emotional-oriented, less beneficial, and ineffective coping mechanisms. There was no correlation between both disorders and burden [34].

Patients who have been diagnosed with bipolar disorder or schizophrenia participated in a cross-sectional survey with matched caregivers. In a survey of 297 caregivers, it was discovered that the individual’s diagnosis of SCZ or BPD had no bearing on the degree of caregiver load [35].

Data on relatives of people with SCZ and BPD were published in two studies as part of a larger cohort. The studies have highlighted the possibility that, despite the perception that bipolar illnesses are episodic, both diagnoses may carry a similar cost due to the greater chronicity and intensity of symptoms across both of them. Also in these analytical studies, the authors contrasted the stress on caregivers of BPD patients and those with schizophrenia. There were 450 family relatives in total, and it was discovered that the burden of both diseases on caretakers was equal [36,37].

A total of 52 older patients with BPD and SCZ with their family caregivers took part in a cross-sectional study to gauge the perceived load. The researchers discovered that the mental QOL of caregivers deteriorated, with a mean mental score of 59.05; 42.3% of caregivers evaluated their perceived strain to be excessive at a threshold of 17. Age- and gender-specific demographic factors, as well as patient health-associated factors (dependency level, age at illness onset and length of clinical remission), were taken into account, and the results revealed that depression level and stress in caretakers significantly influenced how much of a burden they felt. The caretakers' burden did not significantly differ among both groups [38].

In both groups, caregiver traits such as lower educational attainment, being a spousal caregiver, and more caregiver distress were linked to burden.

Depression and altered QOL

In accordance with the World Health Organization, QOL is defined as "a person's perception of their place in life in respect to their objectives, standards, and issues in light of the culture and value systems they adhere to" [39]. It is well established that those who care for individuals with mental illness are at risk for psychological issues like sadness, and anxiety [40]. A study done on Arab people revealed that the QOL was significantly decreased when anxiety and depression coexisted [41]. Wong et al. came to the conclusion that the strain of looking after a family member who has a mental illness may lower the caregiver's QOL. Mental and physical health are inextricably linked [42].

Studies have indicated that increased levels of stress, depression, anxiety and insomnia among caregivers were connected with a decline in mental and physical QOL. Living with a mentally ill relative who has SCZ has been connected to greater levels of anxiety, depression in various studies [43-45], and bipolar disorder [46]. Long-term family caregivers of SCZ patients experienced high levels of anxiety and sadness in addition to poor physical QOL [47]. In consonance with prior studies, severe depressive symptoms were present in family caregivers of individuals with mental illness, which had an adverse effect on their physical QOL [48]. More than half of those who cared for people who had severe mental illnesses displayed extremely low QOL. These results can be seen as a clear illustration of how the healthcare system has failed to support family caregivers in meeting their needs and taking care of their ongoing worries [49-52].

In a cross-sectional study of 186 caretakers of SCZ patients, it was discovered that the majority of them had depressive symptoms and that three-fifths of them had high levels of emotional empathy. There were significant positive connections between the caregivers' overall levels of burden and their levels of sadness and emotional empathy. Additionally, there was a significant inverse relationship between the caregivers' overall degrees of emotional empathy and their depression levels [53].

In a prospective study conducted in Sri Lanka, it was discovered that nearly 40% of the caregivers in the study screened positively for signs of depression and that almost 50% expressed dissatisfaction with their lives, despite the fact that they never sought out formal counselling sessions for their issues despite acknowledging the need for the sessions to relieve their mental stress. Spending more time with the patients and experiencing violence was another big risk factor for depression in caregivers. Additionally, factors more likely to have an impact on the caregiver's current living situation were linked to lower levels of life satisfaction [54].

According to a study in the United States [55], the stress on several facets of patient behaviour among caregivers of BPD patients ranged from 50% to 80%. Higher burden bearers also had a higher likelihood of experiencing depressed symptoms [56]. Another cross-sectional study of 157 Taiwanese primary family caregivers of schizophrenia patients found that older and less educated caregivers experienced a higher caregiver burden and lower QOL [57].

In a cross-sectional study with 100 patients to evaluate the QOL of family members of people with BPD, it was discovered that age is a determinant of QOL and that the gender of the patient had no effect on QOL [58]. However, another study was done on 77 people to determine the level of caregiving load among spouses of BPD patients. This study included 44 spouses of BPD I patients and 33 spouses of BPD II patients. The study's findings indicated that men accepted wife's mental illness more frequently than women and that women, on average, were more burdened with patient care. Men also accepted wives' mental illnesses more frequently than women [59].

Greater burden on caregivers of BPD patients

The caring load was substantially more in BPD caregivers in contrast to those of SCZ, according to two studies with a combined sample size of 565 caregivers (7.7%). According to Zhou et al., caregivers of people with acute BPD had higher perceptions of aggressive behaviour (B = 2.01), and risk of suicide (B = 0.5) than those of people with acute SCZ spectrum disorders. Although focusing on acute illness presentations may elucidate the results, severity comparison of symptoms was not feasible due to variations in the measured symptoms they utilised. According to the involvement assessment questionnaire (IEQ) (t = 2.96), the author found that caregivers of people with BPD rated their burden as being higher than people with caretakers of SCZ. According to the author's analysis of the IEQ (t = 2.96), caregivers of people with BPD perceived their caregiving load as being greater than that of those with SCZ [60,61]. Only these two studies state that BPD caregivers experience a higher burden than SCZ.

## Conclusions

Although people with mental illnesses are directly impacted, their caretakers are also negatively impacted. Both the clan with SCZ and those who have BPD bear a heavy load. However, many studies showed that caregivers of patients with SCZ carried a heavier load. The caregivers should have enough help to keep their own mental health in check. Support for sustaining their mental health should be given to them. By prescribing caregiver checkups and assessments during their families' normal appointments with doctors, healthcare professionals can significantly reduce the levels of caregiver sadness and anxiety. Therefore, in order to encourage caregivers of psychologically ill people and meet their psychological needs, the promotion of mental health is crucial. This can be done by fostering social assistance and respect is prioritised in an inclusive setting.
